# Deconstructing Therapeutic Failure with Inhaled Therapy in Hospitalized Patients: Phenotypes, Risk Profiles, and Clinical Inertia

**DOI:** 10.3390/biomedicines13122892

**Published:** 2025-11-26

**Authors:** Myriam Calle Rubio, Soha Esmaili, Juan Luis Rodríguez Hermosa, Iman Esmaili, Pedro José Adami Teppa, Miriam García Carro, José Carlos Tallón Martínez, Ángel Nieto Sánchez, Consolación Riesco Rubio, Laura Fernández Cortés, María Morales Dueñas, Valeria Chamorro del Barrio, Xinyi Gao

**Affiliations:** 1 Pulmonology Department, Hospital Clínico San Carlos, Instituto de Investigación Sanitaria del Hospital Clínico San Carlos (IdISSC), 28040 Madrid, Spain; mcallerubio@gmail.com (M.C.R.); adamipedro92@gmail.com (P.J.A.T.); valeria.chamorro@salud.madrid.org (V.C.d.B.); xygao2112@gmail.com (X.G.); 2 Department of Medicine, School of Medicine, Universidad Complutense de Madrid, 28040 Madrid, Spain; soha@esmaili.ws (S.E.);; 3 CIBER de Enfermedades Respiratorias (CIBERES), 28029 Madrid, Spain; 4 Pulmonology Department, Hospital Universitario La Zarzuela, Hospital Quirónsalud San Jose, 28040 Madrid, Spain; 5 Heart Lung Innovation Centre, Vancouver, BC V6Z 1Y6, Canada; 6 School of Medicine, Universidad Antonio de Nebrija, 28029 Madrid, Spain; 7 ISNS Data Analytics and Research, Vancouver, BC V6B 1J6, Canada; iman@esmaili.ca; 8 Farmacy Department, Hospital Clínico San Carlos, 28040 Madrid, Spain; 9 Internal Medicine Departament, Hospital Clínico San Carlos, 28040 Madrid, Spain

**Keywords:** therapeutic failure, clinical phenotypes, clinical inertia, inhaled therapy, hospitalized patients, inhaler technique

## Abstract

**Background**: Hospitalized patients on chronic inhaled therapy suffer high rates of therapeutic failure. Current approaches often overlook patient heterogeneity, treating failure as a uniform problem. We hypothesized that clinical inertia, a key driver of failure, is not a monolithic entity but is governed by specific, non-overlapping factors. **Methods**: In this unicentric, observational cohort study of 499 hospitalized adults on chronic inhaled therapy, we used unsupervised clustering to identify patient phenotypes. Multivariable logistic regression was used to model predictors of critical inhaler errors and three distinct forms of clinical inertia: Therapeutic Class (TCI), Device-Level (DLI), and Adherence-Related (ARI). **Results**: Inhaler misuse was driven by objective capability—deficient knowledge (aOR 6.03, 95% CI 2.88–12.64) and low inspiratory flow (aOR 3.11, 95% CI 1.06–9.12)—while patient-reported adherence was not a significant independent predictor. Crucially, the three forms of clinical inertia were governed by distinct, non-overlapping predictors: TCI was predicted by high therapeutic potency (aOR 7.80, 95% CI 3.65–16.64), DLI by a failure in the clinical process (lack of patient training, aOR 3.49, 95% CI 1.21–10.03), and ARI by regimen complexity (aOR 0.06, 95% CI 0.02–0.25). Post-discharge mortality (21.6% overall; 25.8% in Cluster 1 vs. 18.3% in Cluster 2) was independently predicted by objective risk factors, including older age (aOR 1.51, 95% CI 1.20–1.89) and an unassessed inspiratory flow (aOR 2.44, 95% CI 1.19–5.03). Two underlying patient phenotypes were identified—an “Unassessed/Older” (n = 225) and an “Assessed/Younger” (n = 274)—which represented distinct in-hospital care pathways but did not independently predict mortality after multivariate adjustment. **Conclusions**: Therapeutic failure in hospitalized patients is a predictable outcome driven by distinct, non-overlapping factors. This study deconstructs this failure by identifying the specific, actionable drivers of inhaler misuse (patient capability) and the three forms of clinical inertia (therapeutic potency, failures in the care process, and regimen complexity). These processes occur within two distinct patient phenotypes that represent different in-hospital care pathways. Our findings provide a new framework to move beyond generic interventions toward a more precise, evidence-based approach to inhaled therapy.

## 1. Introduction

Chronic respiratory diseases impose a substantial global burden of morbidity and mortality, representing a leading cause of disability and escalating healthcare expenditure [1]. Effective inhaled therapy remains the cornerstone of their management, designed to deliver medication directly to the lungs while minimizing systemic exposure [2]. However, a persistent and profound gap exists between the potential efficacy of these treatments demonstrated in controlled trials and their real-world effectiveness. This disparity is a primary factor in poor clinical outcomes, fueling a vicious cycle of preventable exacerbations, hospital readmissions, and increased mortality [3].

A key mechanism underlying this therapeutic failure is the alarmingly high prevalence of critical errors in inhaler technique. Such errors, which severely compromise or negate drug delivery, are observed in a significant proportion of patients, with rates that can exceed 50% for certain devices [4]. These technical deficiencies are not benign omissions; they are directly correlated with worsened disease control, increased reliance on rescue medications, and a higher frequency of costly hospitalizations [5]. The inpatient setting represents a high-stakes paradox in this context: it offers a key opportunity to identify, evaluate, and correct these life-altering deficiencies. Yet, it is precisely in this environment—where patients are at their most vulnerable—that this opportunity is often systematically missed, perpetuating a cycle of therapeutic failure [6].

Despite these high stakes, the etiology of therapeutic failure in hospitalized patients remains incompletely characterized. Current explanations are often reductionist, with core components—such as patient-level factors like poor adherence [7] and system-level factors like clinical inertia (the failure of clinicians to act when indicated [8])—typically investigated in separate research silos [9]. This fragmented perspective has treated clinical inertia as a monolithic problem, fostering a “one-size-fits-all” approach to intervention that ignores the profound heterogeneity among patients [10]. This inertia is not theoretical; evidence from clinical practice shows that only a small fraction of patients had their inhaler changed at discharge, despite widespread evidence of incorrect use. Evidence suggests a systemic bias where clinical decisions are driven more by pharmacological adjustments than by a documented mismatch between the device and the patient’s inspiratory capacity [11]. The critical blind spot in current practice is the absence of a robust framework to identify which patients are prone to which specific types of failure. This knowledge gap is particularly pronounced in the complex, multimorbid elderly population where these challenges are most acute and the consequences most severe [12].

To deconstruct this complexity and move beyond single-factor explanations, the present study was designed to apply a multidimensional, data-driven approach. We hypothesized that this would allow us to identify distinct, clinically meaningful phenotypes of hospitalized patients. We posit that each phenotype possesses a unique profile of risks and actionable predictors of therapeutic failure, thereby paving the way for targeted, personalized interventions. We sought to create an integrated model that connects patient characteristics, objective functional capacity, and specific, quantifiable patterns of clinical inaction.

To achieve this, the study’s objectives were to: (1) identify these distinct clinical phenotypes based on patients’ inhaler use profiles and clinical characteristics; (2) determine the independent predictors of three specific forms of clinical inertia—Therapeutic Class, Device-Level, and Adherence-Related; (3) compare 90-day mortality across the identified phenotypes; (4) synthesize the drivers of failure across a dynamic spectrum of patient functional capacity; and (5) explore whether the patient phenotype modifies the effect of key clinical risk factors. Through this comprehensive approach, we aimed to establish a new, evidence-based framework for personalizing the management of inhaled therapy in the acute care setting.

## 2. Materials and Methods

### 2.1. Study Design and Setting

This was a pre-specified analysis of a prospectively enrolled cohort from the AIRE (Real-World Evidence on the Use of Inhalers) project, a unicentric, observational study conducted from March 2023 through March 2024 at the Hospital Clínico San Carlos, a large tertiary university hospital in Madrid, Spain [13]. All participants provided written informed consent prior to enrollment.

### 2.2. Study Population and Eligibility Criteria

The source population consisted of all adult patients (≥18 years) admitted to medical inpatient services who were prescribed any form of inhaled therapy. For the present analysis, the cohort was restricted to patients with a documented history of chronic inhaled therapy prior to the index hospitalization and for whom a complete, structured in-hospital assessment of their inhaler use was performed. Patients with severe cognitive impairment (operationally defined as a clinical state, documented by the treating team, that precluded the ability to provide informed consent or to understand and perform the standardized inhalation maneuvers) were excluded. Application of these criteria yielded a final analytical cohort of 499 patients.

### 2.3. Data Collection and Measurement

Data were derived from two complementary sources: retrospective extraction from the institutional electronic health record (EHR) and a prospective, structured, in-person assessment during the index hospitalization. To mitigate observation bias and ensure the study captured an unbiased reflection of routine clinical practice, the treating medical and nursing teams remained strictly blinded to all research-specific assessments and their results. This structural blinding was maintained because the prospective assessment was performed by a dedicated, research-only team of respiratory nurses (detailed below) who were separate from the patient’s treating team and instructed not to discuss their research findings with the clinical staff.

Retrospective data included patient demographics, primary admission diagnosis, and comorbidities quantified using the validated Charlson Comorbidity Index [14]. A comprehensive pharmacological history was ascertained by cross-referencing regional and in-hospital prescription databases. The primary clinical outcome, 90-day all-cause mortality, was determined via the EHR’s link to the Spanish national death registry (SIP-CIBELES).

The prospective assessment was performed by a dedicated team of respiratory nurses and comprised a single, structured evaluation that included: (1) Peak Inspiratory Flow (PIF) measurement using a calibrated In-Check DIAL G16 device; (2) Inhalation technique evaluation using device-specific checklists to identify critical errors; (3) Adherence assessment using the validated 10-item Test of Adherence to Inhalers (TAI) [15]; and (4) Patient-reported knowledge of inhaler handling evaluated using a 5-point Likert scale (from −2: very poor to +2: Excellent). For analytical purposes, this scale was subsequently categorized into three levels: Poor (scores −2 and −1), Fair (score 0), Good (score +1 and +2).

### 2.4. Variable Selection and Operational Definitions

All exposure and outcome variables were prospectively defined to ensure methodological rigor and baseline predictors were obtained at admission, process predictors during the structured in-hospital evaluation, and inertia outcomes (TCI, DLI, ARI) were determined exclusively at discharge.

- Primary Exposure: The primary exposure was the patient phenotype, which was derived empirically from the data using the unsupervised cluster analysis detailed in Section 2.6.
- Primary Patient-Level Outcome: The main clinical outcome was the presence of at least one device-specific critical error, identified via validated checklists.
- Process-of-Care Outcomes (Clinical Inertia): Three distinct, non-mutually exclusive forms of clinical inertia were defined. These domains were selected a priori to capture the three complementary mechanisms of suboptimal care: behavioral barriers (Device-Level), therapeutic decision-making (Therapeutic Class), and regimen implementation (Adherence-Related).

(a) Device-Level Inertia (DLI): Defined as the failure to change the inhaler device type at discharge for a patient with a documented, device-specific critical inhalation error with their home device. This unbiased, technique-focused definition was used to ensure a methodologically consistent comparison of clinical inertia across all device classes.
(b) Therapeutic Class Inertia (TCI): Defined as the failure to escalate inhaled therapy at discharge for a high-risk patient. High-risk status was determined by evidence of significant healthcare resource utilization in the preceding year (≥1 respiratory-related hospitalization or emergency department visit, or ≥2 courses of systemic antibiotics).
(c) Adherence-Related Inertia (ARI): Defined as the failure to simplify the inhaler regimen (e.g., reducing the number of devices) at discharge for a patient with documented poor adherence, defined as a Test of Adherence to Inhalers (TAI) score < 50.

- Composite indicators: Finally, to quantify the patient’s objective capability to use their device, a composite Clinical Frailty and Competence Score (CFCS) was developed a priori. As detailed in the Methodological Analysis—Supplementary Materials File S2 (Section G), this score was constructed by averaging three rescaled domains directly related to inhaler use: (a) peak inspiratory flow, (b) a knowledge composite, and (c) TAI adherence. The score was normalized to a 0–1 scale (higher values = greater capability). The robustness of the CFCS as a predictor was validated through extensive sensitivity analyses (also provided in Supplementary Materials Section G, Table S10).

To address potential temporal ambiguity, the timing of all measurements was pre-specified to ensure predictors were measured chronologically before outcomes. Baseline predictors (e.g., age, comorbidities, pre-admission device) were extracted from the admission record. Process predictors (e.g., PIF, handling knowledge) were collected during the single, structured in-person assessment during the index hospitalization. All three inertia outcomes (TCI, DLI, ARI) were ascertained at the point of hospital discharge.

### 2.5. Sample Size and Power Considerations

The analytical cohort comprised 499 consecutive patients who met all eligibility criteria during the one-year study period. A formal a priori sample size calculation was not performed, which is a standard approach for studies with exploratory primary objectives, such as the data-driven identification of clinical phenotypes. However, the resulting sample size was robust, providing sufficient statistical power to ensure the stability of the multivariable regression models. For all primary outcomes, the cohort size satisfied standard methodological criteria, including the events-per-variable ratio, thus minimizing the risk of model overfitting.

### 2.6. Statistical Analysis

All statistical analyses were performed using R, version 4.2.2, with a two-sided *p*-value < 0.05 considered statistically significant. Analyses were conducted on a complete-case basis. This approach was deemed appropriate given the low proportion of missing data (<5%) for the primary variables and assuming that these data were missing completely at random. A crucial distinction in this analysis is the separation of methodological missing data from the clinical finding of ‘failure to assess.’ As detailed in the Methodological Analysis—Supplementary Materials (Section B, Table S5), true missing data for primary variables was minimal (e.g., 1.2% for PIF, 0.8% for TAI). Therefore, the ‘Unknown’ category (e.g., 36.5% for Peak Flow) was not treated as missing data but as a pre-specified, clinically meaningful analytical category representing this failure to assess and real-world non-assessment rather than missingness. This a priori design choice defines the ‘unassessed’ state as a core exposure variable, thus avoiding the potential for circular reasoning.

Furthermore, to validate the robustness of this analytical approach, a sensitivity analysis using multiple imputation was performed (as detailed in Supplementary Materials Section D, Table S7), which confirmed the high stability (97.2% agreement) of the phenotypes.

The analytical strategy proceeded in three sequential phases.

First (Phenotype Identification): An unsupervised k-means cluster analysis was performed on key baseline clinical and functional variables—including age, comorbidity, peak flow, adherence, and handling knowledge—to identify patient subgroups in a data-driven manner. The optimal number of clusters (k = 2) was determined empirically. As detailed in the Methodological Analysis—Supplementary Materials (Section D, Table S7)—which was included in our original submission—this two-cluster solution was quantitatively validated, showing a strong average silhouette score of 0.68 (vs. 0.51 for k = 3). A stability analysis using multiple imputation further confirmed the robustness of this solution, demonstrating 97.2% agreement in patient classification (Supplementary Materials Section D, Table S7).

Second (Predictive Modeling): A series of multivariable logistic regression models were constructed. Covariates for all models were selected a priori based on established clinical relevance to mitigate the risk of spurious associations. The prognostic significance of the derived phenotypes was assessed in a model for 90-day mortality. Separate models identified predictors of critical inhaler errors. To analyze the drivers of clinical inertia, three distinct models were developed for each inertia outcome (TCI, DLI, and ARI), with each model fitted only on its relevant patient denominator (i.e., the specific subpopulation for whom the clinical action was indicated). Model diagnostics, including variance inflation factors, were assessed to rule out significant multicollinearity. To specifically address the DLI analysis and avoid potential tautology, a two-stage modeling approach was used. First, a baseline predictive model was constructed using only pre-admission characteristics. Second, a separate, expanded model was fitted not for prediction, but as an exploratory analysis to understand the association of in-hospital process variables (such as the in-hospital device choice or documented patient training) with the DLI outcome.

Third (Synthesis and Interaction Analysis): The continuous CFCS (a composite score of age, comorbidity, and functional/cognitive status as detailed in Section 2.4) was used to model risk across the full spectrum of patient capability, generating predicted probability curves for each outcome, derived from logistic regression models that included CFCS as a continuous predictor, stratified by phenotype. To conduct an exploratory analysis of effect modification, pre-specified interaction terms between phenotype and key clinical factors were introduced into the main models. Finally, a complementary analysis was conducted by fitting a multivariable model for the outcome of receiving a device change at discharge to identify factors associated with proactive clinical action.

### 2.7. Ethical Considerations

The study protocol was conducted in accordance with the principles of the Declaration of Helsinki and was approved by the Institutional Review Board of the Hospital Clínico San Carlos (CI:23/069-O_M_NoSP). All participants, or their legally authorized representatives, provided written informed consent prior to enrollment and any study-related procedures. All patient data were de-identified prior to analysis to ensure confidentiality.

## 3. Results

### 3.1. Characterization of Clinical Phenotypes, Therapeutic Inertia, and Prognostic Relevance

An unsupervised cluster analysis identified two distinct clinical phenotypes, detailed in Table 1: the “Unassessed/Older” phenotype (Cluster 1) and the “Assessed/Younger” phenotype (Cluster 2).

The “Unassessed/Older” group was defined by significantly older patients and, most notably, a systematic lack of assessment data regarding peak inspiratory flow, adherence, and handling knowledge. This suggests a clinically under-evaluated patient profile. In contrast, the “Assessed/Younger” group was thoroughly characterized, showing high rates of adequate inspiratory flow and proficient handling knowledge.

The phenotypes also differed in pre-admission device use, with pMDIs predominating in Cluster 1 and DPIs in Cluster 2. Despite these differences, therapeutic inertia was high in both groups, as most patients were discharged on their original device type. A trend toward higher 90-day mortality was also observed in the “Unassessed/Older” phenotype.

**Figure 1:**
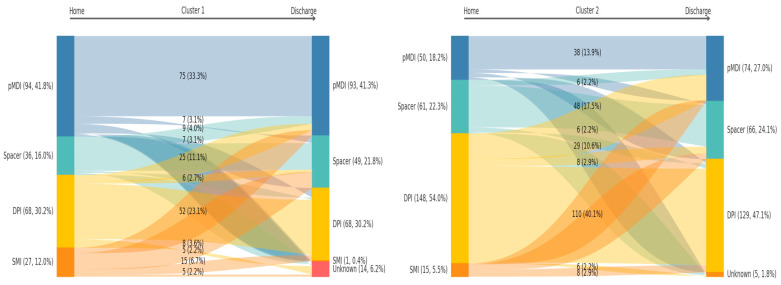
Inhaler device transitions from pre-admission (home) to discharge, stratified by data-driven phenotypes. The Sankey diagrams illustrate the flow of device types from pre-admission (home) to discharge (right) for each phenotype. The height of the bars is proportional to the number of patients (n) and cluster percentage (%). The connecting ribbons represent the transitions, with labels indicating the patient count and cluster percentage for each specific pathway. Abbreviations: DPI, dry powder inhaler; pMDI, pressurized metered-dose inhaler; SMI, soft mist inhaler.

Figure 1 displays the transitions in inhaler device types from pre-admission to discharge, stratified by the two clinical phenotypes. In the “Unassessed/Older-Higher Burden” phenotype (Cluster 1), the most common pathway was patients remaining on a pressurized metered-dose inhaler (pMDI; n = 75), followed by retention of a dry powder inhaler (DPI; n = 52) and a spacer (n = 25). The most frequent device switch was from a soft mist inhaler (SMI) to a spacer (n = 15). In the “Assessed/Better-Documented” phenotype (Cluster 2), retention of a DPI was the most frequent pathway (n = 110), followed by patients remaining on a spacer (n = 48) and a pMDI (n = 38). The most notable device switch involved patients moving from a DPI to a pMDI (n = 29).

To isolate the independent prognostic value of the phenotype from other known risk factors, we constructed a multivariable logistic regression model for 90-day mortality (Table 2). The adjusted analysis sought to identify the true drivers of mortality in this cohort. After adjusting for potential confounders, the patient phenotype was not a statistically significant independent predictor of mortality, although the “Assessed/Younger” phenotype (Cluster 2) showed a borderline protective association. The analysis revealed that mortality risk was more strongly shaped by established patient factors and specific process-of-care indicators. Older age and a higher comorbidity burden were both associated with a significant increase in the odds of death.

Notably, the inability to measure peak inspiratory flow (“Unknown”) was a powerful predictor of mortality, conferring a greater risk than a known low flow. However, the most striking finding was that the absence of a confirmed diagnosis of documented respiratory comorbidity emerged as the single strongest predictor of death, increasing the odds nearly fourteen-fold compared to patients with a COPD diagnosis (aOR 13.75).

### 3.2. Deconstruction of Therapeutic Failure: Predictors of Inhaler Misuse

Having defined these clinical phenotypes, the next logical step was to determine if the drivers of failure were uniform or specific to each group. To design effective interventions, it is essential to understand the root causes of inhaler misuse. We analyzed how critical error (CE) prevalence deviates from the baseline within each phenotype when stratified by key clinical factors.

**Figure 2:**
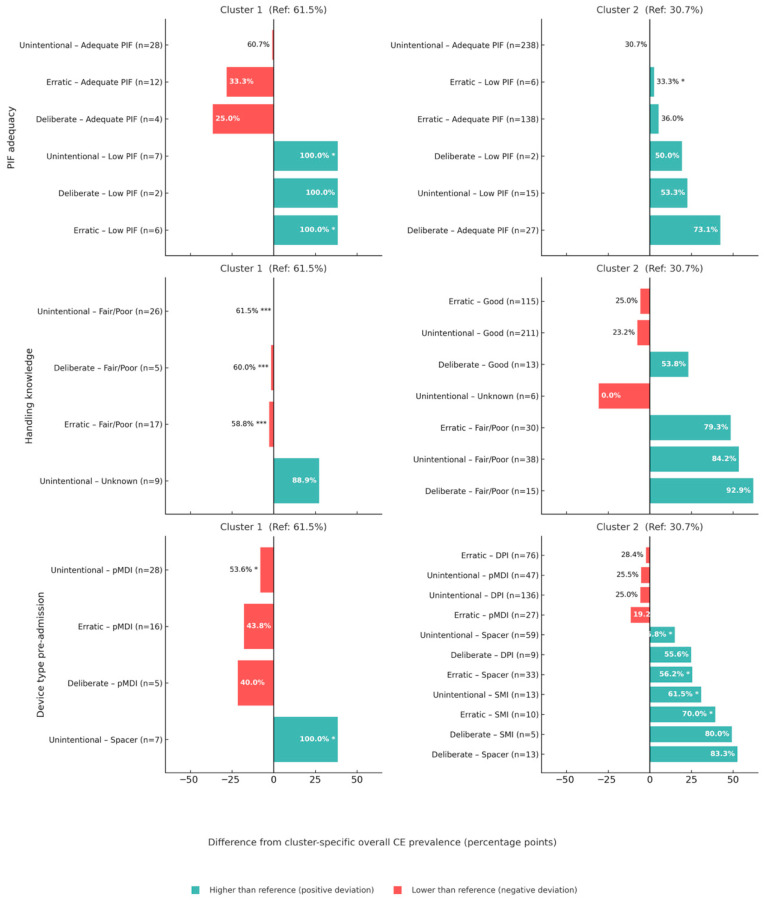
Deviations in Critical Error (CE) Prevalence Within Phenotypes, Stratified by PIF Adequacy, Inhaler-Handling Knowledge, and Pre-Admission Device Type. Bar length and bar labels represent two distinct metrics. The bar length (*x*-axis) indicates the deviation from the cluster-specific reference rate (in percentage points). The bar label (e.g., ‘60.7%’) indicates the absolute critical error (CE) prevalence within that subgroup. Red bars denote a prevalence lower than the reference. Subgroup sample sizes (n) are shown on the *y*-axis. Significance levels are denoted as: *p* < 0.05, * *p* < 0.01, *** *p* < 0.001.

Figure 2 illustrates how the prevalence of critical errors deviates from the cluster-specific baseline across various subgroups. In the “Unassessed/Older” phenotype (Cluster 1), the combination of erratic non-adherence and a low peak inspiratory flow (PIF) was associated with a substantial excess risk of errors (*p* = 0.012). In contrast, adequate PIF within this same subgroup did not significantly alter the baseline risk (*p* = 0.41). Poor handling knowledge was a consistently strong predictor, significantly increasing the likelihood of errors (*p* < 0.001). Regarding devices used prior to admission in this subgroup, all patients who used an inhalation chamber (n = 7) made critical errors; however, this difference was not statistically significant. Furthermore, an analysis of the overall functional capacity score showed it was not significantly associated with critical errors in this phenotype (Supplementary Table S1).

Similar patterns were observed in the “Assessed/Younger” phenotype (Cluster 2), where the high-risk combination of erratic non-adherence with low PIF also resulted in an elevated error rate (*p* = 0.026). Consistent with the findings in Cluster 1, adequate PIF in this subgroup was not associated with a significant change in risk (*p* = 0.37). Deficient handling knowledge remained a significant predictor of misuse (*p* = 0.002). Regarding the effect of the type of device, in Cluster 2 “Assessed/Younger” phenotype, pre-admission use of a Spacer was associated with a strong trend towards an increased risk of errors (*p* = 0.077), and pre-admission use of a DPI was associated with a modest, non-significant reduction in errors (*p* = 0.11). In this phenotype, a higher overall functional capacity score was significantly associated with a lower likelihood of committing critical errors (Supplementary Table S1).

To disentangle the fundamental drivers of inhaler misuse from patient-reported behaviors, we constructed a multivariable model to identify the independent predictors of critical errors after accounting for physiological capacity and handling knowledge. The results of this definitive model are shown in Table 3.

The analysis revealed that after adjusting for all factors, only two variables remained strong, independent predictors of committing a critical inhaler error. Deficient handling knowledge was the most powerful predictor, associated with a six-fold increase in the odds of a critical error. A low peak inspiratory flow (PIF ≤ 30 L/min) was also a significant predictor, more than tripling the odds of error. The pre-admission use of a Spacer was associated with a trend toward an increased risk of errors, although it did not reach statistical significance (*p* = 0.077). Notably, in contrast to their strong unadjusted associations (Supplementary Table S2), once patient knowledge and physical capacity were accounted for, self-reported non-compliance behaviors were not statistically significant predictors of incorrect use.

The identification of objective, correctable problems such as critical errors necessitates clinical intervention. To understand the factors that impede such action, we first explored descriptive variations in inertia prevalence across key clinical subgroups (Supplementary Figure S1) before conducting three separate multivariable analyses to identify the independent predictors of clinical inertia within distinct domains: therapeutic class (TCI), device selection (DLI), and regimen simplification (ARI). As shown in Table 4, each analysis revealed a unique clinical decision pathway with different actionable levers. For Therapeutic Class Inertia (TCI), several factors were significant predictors of inaction. While older age was found to be strongly protective (aOR 0.60, *p* < 0.001), a higher comorbidity burden (aOR 1.90, *p* = 0.003) and admission to a service other than Internal Medicine or Pulmonology (aOR 5.88, *p* = 0.031) were associated with increased inertia. However, higher baseline therapy potency remained the dominant predictor (aOR 7.80, *p* < 0.001). For Adherence-Related Inertia (ARI), a greater number of home maintenance inhalers was strongly protective against inertia (aOR 0.06, *p* < 0.001). For Device-Level Inertia (DLI), which was analyzed using an unbiased, technique-focused definition, the baseline model showed that no single pre-admission device type was a statistically significant predictor. However, a subsequent expanded model that included clinical process variables revealed that the strongest predictor of DLI was a failure in patient care: patients who did not receive training on their inhaler had 3.5-fold higher odds of inertia (aOR 3.49, *p* = 0.020).

### 3.3. Synthesis of Dynamic Risk, Effect Modification, and Levers for Corrective Action

Finally, to integrate these isolated predictors into a cohesive model of patient vulnerability, we synthesized the drivers of misuse and inertia by examining their relationship with a composite Clinical Frailty and Competence Score (CFCS), a continuous measure reflecting overall patient capability. The analysis revealed that the risk profile of patients shifts dramatically across the spectrum of functional capacity, and that these shifts differ by phenotype.

**Figure 3:**
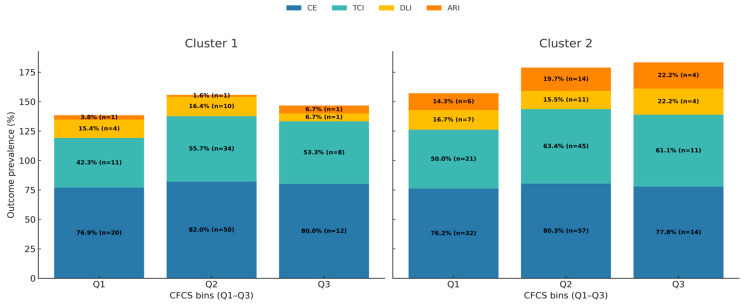
Stacked prevalence of actionable therapeutic failures by CFCS quantiles, stratified by Cluster. The Composite Clinical Frailty and Competence Score (CFCS) was divided into quantile-based bins (Q1–Q3). To improve clarity and simplify the visualization, this figure presents the four actionable process outcomes (CE, TCI, DLI, ARI). 90-day mortality data is presented in Table 2. Outcomes are non-mutually exclusive (a patient can have both CE and TCI); therefore, the total stacked prevalence may exceed 100%. Each bar represents the observed prevalence (%) and absolute number (n) of these outcomes within a CFCS bin, stacked to illustrate their distribution and overlap.

Figure 3 shows the stacked prevalence of adverse outcomes across quantiles of the Composite Clinical Frailty and Competence Score (CFCS), revealing how the risk profile shifts with changes in patient functional capacity. Further details on the prevalence and event counts for each outcome are provided in Supplementary Table S3. A key finding is that Critical Errors (CEs) represented the dominant adverse outcome across all functional levels in both phenotypes, with a prevalence that consistently exceeded 75%. Therapeutic Class Inertia (TCI) was the second most common issue, particularly in the “Assessed/Younger” phenotype, where it affected over 60% of patients in the highest functional group. This indicates a high burden of therapeutic failure that is not uniformly mitigated by higher patient capability.

**Figure 4:**
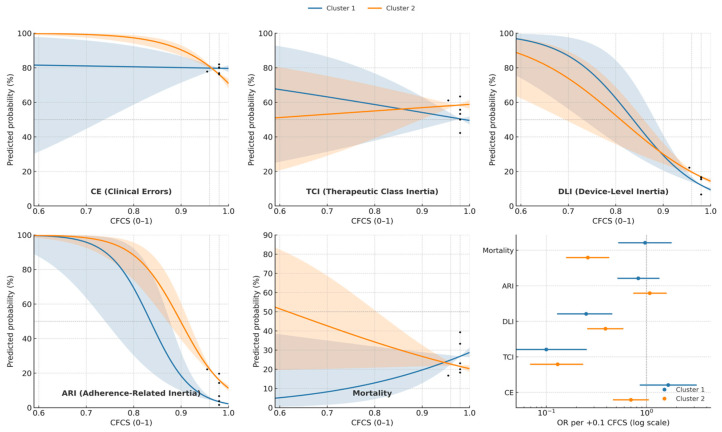
Predicted probability of adverse outcomes across the CFCS spectrum, stratified by Cluster. Curves represent fitted logistic regression models with shaded 95% confidence interval (CI) bands for each outcome: Clinical Errors (CEs), Therapeutic Class Inertia (TCI), Device-Level Inertia (DLI), Adherence-Related Inertia (ARI), and 90-day Mortality. The Composite Functional Capacity Score (CFCS) is scaled from 0 to 1. Probabilities are adjusted to show the expected prevalence across the CFCS spectrum for each phenotype.

Figure 4 presents the modeled probabilities of five adverse outcomes across the full spectrum of the Composite Clinical Frailty and Competence Score (CFCS) for each phenotype. The specific odds ratios for this analysis are detailed in Supplementary Table S1. In both phenotypes, a higher functional capacity was associated with a strong and significant decline in the probability of both Device-Level Inertia (DLI) and Adherence-Related Inertia (ARI). In contrast, for the “Assessed/Better-Documented” phenotype (Cluster 2), improved functional capacity was also strongly protective against Clinical Errors (CEs), an effect not observed in Cluster 1. The risks of TCI and mortality did not show a statistically significant change across the functional spectrum in either phenotype.

Beyond identifying main effects, we performed an exploratory analysis to determine if the patient phenotype modified the relationship between key clinical factors and outcomes. The analysis revealed significant interactions, indicating that the two phenotypes exhibit distinct clinical dynamics. For example, better self-reported adherence was strongly protective against critical errors, but this effect was only present in the “Assessed/Better-Documented” phenotype, while a history of frequent healthcare utilization was associated with fewer errors only in the “Unassessed/Older-Higher Burden” group (Supplementary Figure S2).

This complementary analysis identified the factors associated with a proactive device change at discharge (Table 5). Pre-admission device type was a strong determinant of action. Notably, patients who entered the hospital using a pMDI or SMI were significantly more likely to have their device changed compared to those using a DPI (aOR 1.85, *p* = 0.004). The in-hospital therapeutic pathway was also highly influential. Patients who received only an SMI during their hospitalization had higher odds of a device change, while those who received only a DPI or a Spacer were significantly less likely to have their device changed compared to patients receiving only nebulized therapy.

## 4. Discussion

This study challenges the conventional view of therapeutic failure in hospitalized patients, reframing it not as a random event but as a predictable consequence of a systemic mismatch between patient capability and the clinical system’s capacity to assess and adapt. Our findings, based on identifying distinct clinical phenotypes and deconstructing the predictors of clinical inaction, provide a new, evidence-based framework for understanding and preventing these common adverse events.

### 4.1. Patient Phenotypes as Proxies for In-Hospital Care Pathways

A key finding is the data-driven identification of two patient phenotypes that function as more than simple demographic labels (Table 1). We propose that the “Unassessed/Older” and “Assessed/Younger” clusters represent two distinct pathways of in-hospital care. The “Assessed/Younger” phenotype follows a pathway of active evaluation, where objective metrics of patient performance are collected in line with best practices [16]. In stark contrast, the “Unassessed/Older” phenotype reveals a pathway of clinical omission, where a patient’s perceived frailty or age may lead clinicians to deprioritize objective functional assessment, a known challenge in the acute care of older adults [17].

Critically, and in line with our a priori analytical strategy (Section 2.6), the absence of functional data in this latter group should not be seen as a methodological limitation but rather as a central finding of this study. The “unassessed” state is itself a marker of a high-risk clinical approach. This assertion is substantiated by our multivariable mortality analysis (Table 2), where the inability to measure peak inspiratory flow was a more potent predictor of death than a known, measured deficiency. This demonstrates that the failure to assess is a clinically meaningful variable that likely captures a constellation of unmeasured factors related to frailty and systemic vulnerability not fully encapsulated by standard comorbidity indices [18] or conventional frailty scales [19]. Furthermore, it is worth noting the seemingly paradoxical finding that patients treated with inhaled therapies but without a confirmed diagnosis of respiratory disease had the highest risk of mortality. Rather than suggesting underdiagnosis, this finding likely reflects the frequent use of inhaled therapies for symptomatic relief in high-risk patients admitted with multifactorial respiratory distress [20]. As noted, these are often complex patients with serious underlying non-respiratory conditions (e.g., advanced heart failure), where the high mortality is driven by the acute systemic illness rather than the respiratory condition itself. In this context, the prescription of inhalers acts as a marker of symptomatic dyspnea in a critically vulnerable population, consistent with the ‘case-mix’ complexity highlighted in similar cohorts.

Our results showed a similar paradoxical finding emerged regarding patient knowledge, where poorer handling knowledge appeared to be protective against mortality (Table 2). This result is likely not causal but rather an indicator of confounding by severity. It is plausible that patients with more severe or complex underlying diseases receive more intensive specialist care, including more thorough training on their inhaler devices, leading them to be classified as having “Good” knowledge. In this context, the higher mortality risk would be driven by their underlying clinical severity, for which patient-reported knowledge may act as an indirect proxy. This finding underscores the complexity of interpreting patient-level factors in acutely ill populations.

### 4.2. The Anatomy of Clinical Inertia: Context-Specific Drivers of Inaction

Our study dissects clinical inertia into three distinct subtypes, revealing that the failure to act is not a uniform behavior but a context-dependent decision influenced by different clinical cues (Table 4).

Therapeutic Class Inertia (TCI) was associated with nuanced, and at times counterintuitive, clinical reasoning. While clinicians were appropriately more proactive with older patients, they exhibited significant inertia in patients with a high comorbidity burden or those already on high-potency regimens. This suggests a “ceiling effect” heuristic, where clinicians may perceive little room for further pharmacological optimization, even when objective risk factors warrant it [21], a phenomenon observed in other chronic conditions [22].

Device-Level Inertia (DLI), in contrast, was not governed by a powerful device-specific bias but rather by failures in the clinical process. After correcting the outcome definition to create an unbiased, technique-focused measure, our multivariable analysis showed that the pre-admission device type was not a significant predictor of clinical inaction (Table 4, Section B.1). To identify the true predictors of this process failure, an expanded analysis revealed that DLI was strongly predicted by a core component of patient care: patients who did not receive training on their inhaler were 3.5 times more likely to be discharged with a device they used incorrectly (aOR 3.49, *p* = 0.020) (Table 4, Section B.2).

This revised understanding is supported by our complementary analysis (Table 5), which showed that patients on a pMDI/SMI were significantly more likely to have their device changed at discharge compared to those on a DPI, reinforcing that no single device class was immune to clinical review. It is crucial to note that our analysis does not only reveal inaction. The device transitions illustrated in Figure 1 also bring to light examples of proactive clinical practice. The most notable device switch in the ‘Unassessed/Older-Higher Burden’ phenotype was from a soft mist inhaler (SMI) to a spacer (n = 15). This transition is clinically highly significant, as it represents a deliberate and evidence-based decision likely made during the in-hospital assessment. However, it should be noted that in the phenotype “Assessed/Younger”, more than 90% of patients had a PIF greater than or equal to 30 L/min and only 54% used DPI, which could reflect a lack of awareness in the decision to verify and adapt to an easier-to-use device. This reinforces that systematic patient assessment—which must consider the full context of device choice, from its environmental footprint and technical demands [23,24] to the patient’s physiological capacity and skill [25]—is the main catalyst for overcoming clinical inertia.

Adherence-Related Inertia (ARI) presented a paradox. Clinicians were highly proactive in simplifying complex regimens for poorly adherent patients—a key guideline recommendation supported by evidence [26]. However, they remained inert to other documented forms of non-adherence. This indicates that while structural barriers (e.g., regimen complexity) are recognized and acted upon, more nuanced behavioral aspects may be systematically overlooked in the acute care setting.

### 4.3. Reframing Inhaler Misuse: It Is Competence, Not Reported Compliance

Our findings challenge the conventional reliance on patient-reported adherence as a primary predictor of effective inhaler use. While various non-adherence patterns were associated with critical errors in unadjusted analyses (Supplementary Table S2), the multivariable model was unequivocal: after accounting for device handling knowledge and physiological capacity (PIF), no other factor remained an independent predictor of misuse (Table 3). The lower error rate observed with pMDIs in the older, unassessed cohort does not necessarily imply the device’s superiority, but may rather reflect greater patient familiarity with this long-established device type.

The implications of this finding are significant. It strongly suggests that patient-reported adherence, while valuable for understanding intent, is a poor proxy for effective drug administration, a known limitation of such measures in adherence research [27]. The critical determinants of whether the prescribed drug reaches the lungs are what a patient knows (technique) and what their body can do (generate sufficient inspiratory flow). This suggests that direct observation of technique and objective PIF measurement are indispensable cornerstones of inhaler therapy assessment, displacing the reliance on patient self-report [28]. This is particularly critical given that the prevalence of critical errors often exceeds 50%, even in stable outpatient populations [29].

### 4.4. A Dynamic Risk Model: How Phenotype Modifies the Relationship Between Function and Failure

This study’s findings support a dynamic model where risk is not a static attribute but is modulated by the interaction between a patient’s functional capacity and their clinical phenotype. By modeling risk across a continuous spectrum of capability (the CFCS), we observed that the trajectory of risk is fundamentally altered by a patient’s care pathway. This approach of modeling risk dynamically is gaining traction for managing other complex chronic diseases [30].

For the “Assessed/Younger” phenotype, improved functional capacity was, as expected, broadly protective against adverse outcomes (Figure 4). In the “Unassessed/Older” phenotype, however, the protective effect against critical errors was absent, with no significant association observed between functional capacity and the risk of misuse.

Furthermore, the significant interaction effects observed (Supplementary Figure S2) provide strong statistical evidence that the predictors of therapeutic failure operate heterogeneously across phenotypes. This finding dismantles the notion of a “one-size-fits-all” intervention. It strongly indicates that to be effective, strategies must be stratified and tailored to the unique risk dynamics of the patient subgroup, a core tenet of personalized respiratory medicine [31].

### 4.5. Limitations

This study has several limitations that warrant consideration. First, the single-center design may limit the generalizability of our findings; however, the setting in a large, tertiary urban hospital with a diverse patient cohort enhances their potential relevance. Second, as an observational study, our findings represent associations and cannot establish causality. We sought to mitigate this through rigorous prospective data collection by a dedicated, blinded research team, a design feature intended to reduce the risk of both information and observation bias.

Third, while our multivariable models controlled for a wide range of covariates, including a validated comorbidity index and a novel frailty score (CFCS), the potential for residual confounding remains. For instance, although severe cognitive impairment was an exclusion criterion, the differential prevalence of mild-to-moderate cognitive decline between phenotypes was not formally assessed and may act as an unmeasured confounder. This is particularly pertinent to the ‘unassessed’ status. This association could be partly explained by confounding by indication (which introduces potential temporal ambiguity), where clinicians may be less likely to perform functional tests on patients they already perceive as too frail. We argue, however, that this clinical judgment is not merely a confounder but is precisely the mechanism that defines the “Unassessed/Older” phenotype. It is a core component of the phenomenon under investigation rather than a simple methodological limitation. Finally, although our data-driven phenotyping is supported by both empirical metrics and clear clinical interpretability, meeting the standard for such analyses [32], these phenotypes require validation in external cohorts.

Finally, while our primary predictive models were designed to test distinct, pre-specified clinical hypotheses, we acknowledge that the multiple exploratory subgroup analyses (e.g., in Figure 2 and Supplementary Figures S1 and S2) were intended as hypothesis-generating. These secondary findings should therefore be interpreted with appropriate caution, as they were not corrected for multiple comparisons.

### 4.6. Clinical Implications and Future Directions

The implications of our findings are direct and actionable, mandating a shift from generic approaches toward a stratified, phenotype-guided framework for inhaler management. Clinically, the act of not assessing a patient’s functional capacity or inhaler technique should be reclassified from a passive omission to an active marker of risk that demands a higher level of vigilance. This supports the implementation of systematic screening protocols for PIF and inhaler technique for all hospitalized patients on chronic therapy, irrespective of age or perceived frailty. Furthermore, the CFCS offers a potential template for an integrated, bedside risk score that could trigger targeted, phenotype-guided interventions, helping clinicians decide whether to prioritize device selection, technique training, or therapeutic escalation based on the patient’s specific risk profile.

Future research should focus on validating these phenotypes in multicentric cohorts to confirm their stability and transportability. The ultimate goal, however, is to move from description to intervention. Randomized trials are needed to test whether phenotype-specific strategies—such as a “technique-first” intervention for functionally capable but error-prone patients versus a “device-first” approach for the frail—can successfully disrupt the pathways to therapeutic failure and improve patient outcomes. Such pragmatic trials testing care delivery strategies are a recognized research priority [33]. Finally, investigating the mechanisms behind our paradoxical findings will be critical to fully understanding the complex human and systemic factors that influence the success or failure of inhaled therapy.

## 5. Conclusions

In this analysis of hospitalized patients on chronic inhaled therapy, we identified two clinically distinct phenotypes that correspond to divergent pathways of in-hospital assessment and management. Our findings demonstrate that therapeutic failure is not a uniform event but is driven by a specific constellation of measurable factors. Deficient device-handling knowledge and low peak inspiratory flow are the primary determinants of inhaler misuse, superseding the predictive value of self-reported adherence. Furthermore, clinical inertia manifests in context-dependent forms, each with unique predictors related to patient comorbidity, baseline therapy, and failures in the clinical process, such as the lack of patient training. The risk of these failures is dynamic and is significantly modified by the interaction between a patient’s functional capacity and their clinical phenotype.

Collectively, these findings indicate that therapeutic failure in this vulnerable population is a predictable outcome of a systemic mismatch between a patient’s objective capabilities and the clinical team’s capacity to assess and act on them. These results provide a strong rationale for shifting from generic interventions toward a personalized, phenotype-guided framework to mitigate therapeutic failure and improve patient outcomes.

## Figures and Tables

**Table 1:** Baseline Characteristics of the Study Cohort by Phenotype.

Domain/Variable	Overall (n = 499)	Cluster 1 (n = 225)	Cluster 2 (n = 274)	*p*
**Demographics & Comorbidity**				
Age (years)	78.0 [69.0–85.0]	80.0 [70.0–86.0]	76.0 [67.0–84.0]	0.010
Charlson comorbidity index	3.0 [1.0–4.0]	3.0 [1.0–4.0]	2.0 [1.0–4.0]	0.218
Sex				0.046
Female	165 (33.1)	66 (29.3)	99 (36.1)	
Male	243 (48.7)	108 (48.0)	135 (49.3)	
Not recorded	91 (18.2)	51 (22.7)	40 (14.6)	
**Respiratory Function & Adherence**				
Peak flow (L/min)	60.00 [45.00–70.00]	55.00 [40.00–60.00]	60.0 [50.0–70.0]	0.001
Peak flow adequacy				<0.001
≥30 L/min	291 (58.3)	39 (17.3)	252 (92.0)	
Unknown	182 (36.5)	176 (78.2)	6 (2.2)	
<30 L/min	26 (5.2)	10 (4.4)	16 (5.8)	
TAI sum	48.0 [47.0–48.0]	48.0 [48.0–48.0]	48.0 [45.0–50.0]	0.613
Adherence				<0.001
Good	84 (16.8)	2 (0.9)	82 (29.9)	
Intermediate	89 (17.8)	3 (1.3)	86 (31.4)	
Poor	106 (21.2)	16 (7.1)	90 (32.8)	
Unknown	220 (44.1)	204 (90.7)	16 (5.8)	
Handling knowledge				<0.001
Fair/Poor	74 (14.8)	30 (13.3)	44 (16.1)	
Good	226 (45.3)	2 (0.9)	224 (81.8)	
Unknown	199 (39.9)	193 (85.8)	6 (2.2)	
**Device Use**				
Device before admission				<0.001
DPI	216 (43.3)	68 (30.2)	148 (54.0)	
SMI	42 (8.4)	27 (12.0)	15 (5.5)	
Spacer	97 (19.4)	36 (16.0)	61 (22.3)	
pMDI	144 (28.9)	94 (41.8)	50 (18.2)	
In-hospital device				0.575
DPI only	14 (2.8)	7 (3.1)	7 (2.6)	
Neb + pMDI + Spacer	3 (0.6)	0 (0.0)	3 (1.1)	
Neb only	207 (41.5)	99 (44.0)	108 (39.4)	
SMI + pMDI	2 (0.4)	1 (0.4)	1 (0.4)	
SMI only	11 (2.2)	7 (3.1)	4 (1.5)	
pMDI + DPI	3 (0.6)	1 (0.4)	2 (0.7)	
pMDI only	257 (51.5)	109 (48.4)	148 (54.0)	
pMDI + Spacer only	2 (0.4)	1 (0.4)	1 (0.4)	
Device changed at discharge				0.038
No	348 (69.7)	152 (67.6)	196 (71.5)	
Unknown	19 (3.8)	14 (6.2)	5 (1.8)	
Yes	132 (26.5)	59 (26.2)	73 (26.6)	
**Clinical Service & Diagnoses**				
Admitting service (Pulmonology)	123 (24.6)	49 (21.8)	74 (27.0)	0.177
Dementia diagnosis	23 (4.6)	10 (4.4)	13 (4.7)	0.874
Heart failure diagnosis	141 (28.3)	62 (27.6)	79 (28.8)	0.753
**Outcomes**				
90-day mortality	108 (21.6)	58 (25.8)	50 (18.3)	0.055

Note. Values are median [interquartile range] for continuous variables and n (%) for categorical variables. *p*-values are from Mann–Whitney U tests for continuous data and χ^2^ or Fisher’s exact tests for categorical data. Abbreviations: DPI, dry powder inhaler; IQR, interquartile range; pMDI, pressurized metered-dose inhaler; SMI, soft mist inhaler; TAI, Test of Adherence to Inhalers. The ‘Not recorded’ sex category reflects a systematic artifact in the administrative database extraction; this variable was not a primary predictor, and sensitivity analyses confirmed it did not alter model results.

**Table 2:** Multivariable Logistic Regression for 90-Day Post-Discharge Mortality.

Predictor	Level/Contrast	aOR	95% CI	*p*
Phenotype	Cluster 2 vs. 1	0.45	0.20–1.01	0.053
Age	per 10-year increase	1.51	1.20–1.89	<0.001
Sex	Male vs. Female	1.09	0.72–1.65	0.682
Charlson comorbidity index	per point	1.14	1.03–1.27	0.013
COPD diagnosis	Yes vs. No	0.54	0.33–0.91	0.019
Peak flow adequacy	Ref: >30 L/min			
≤30 L/min		1.46	0.53–4.04	0.467
Unknown		2.44	1.19–5.03	0.016
Admitting service	Ref: Internal Medicine			
Pulmonology		1.25	0.70–2.24	0.453
Other		0.57	0.31–1.05	0.071
Knowledge—patient handling	Ref: Good			<0.001
Fair		0.40	0.21–0.76	0.005
Poor		0.20	0.07–0.58	0.004
Very Poor		0.00 *	0.00–0.00	<0.001
Undocumented		0.00 *	0.00–0.00	<0.001
In-hospital device	Ref: Nebulizer only			<0.001
pMDI only		0.25	0.09–0.74	0.016
SMI only		0.10	0.02–0.51	0.007
DPI only		0.41	0.08–2.08	0.283
Mixed/Combo categories		~0.00–0.30	various	<0.05
Respiratory comorbidity	Ref: COPD			<0.001
Asthma		0.44	0.18–1.09	0.077
Bronchiectasis		0.38	0.12–1.20	0.098
Others		0.49	0.19–1.26	0.139
No respiratory comorbidity		13.75	6.20–37.28	<0.001

Note. Values are adjusted odds ratios (aORs) with 95% confidence intervals (CIs). Ref indicates the reference category against which other levels are compared. An aOR < 1.0 indicates lower odds relative to the reference; an aOR > 1.0 indicates higher odds. * Near-zero or extremely large aORs indicate quasi-separation of data due to sparse categories and should be interpreted for the directionality of the effect rather than for the magnitude. Model performance and calibration statistics (e.g., AUC, Hosmer–Lemeshow) are detailed in Supplementary Materials Section E, Table S8.

**Table 3:** Multivariable Logistic Regression Predicting Critical Inhaler Errors.

Predictor	Adjusted Odds Ratio (aOR)	95% Confidence Interval (CI)	*p*-Value
Handling knowledge (worse vs. better)	6.03	2.88–12.64	<0.001
PIF ≤ 30 L/min (vs. >30 L/min)	3.11	1.06–9.12	0.038
Deliberate non-adherence (Yes vs. No)	2.24	0.86–5.87	0.100
Erratic non-adherence (Yes vs. No)	1.02	0.54–1.93	0.943
Pre-Admission Device (Ref: DPI)			
Spacer	2.13	0.89–4.05	0.077
SMI	3.83	0.94–12.45	0.082
pMDI	0.95	0.45–2.02	0.902

Note. Values are adjusted odds ratios (aORs) with 95% confidence intervals (CI). An aOR > 1 indicates higher odds of a critical error; an aOR < 1 indicates lower odds. The model was adjusted for all listed predictors. Abbreviations: CI, confidence interval; DPI, dry powder inhaler; PIF, peak inspiratory flow; pMDI, pressurized metered-dose inhaler; SMI, soft mist inhaler. Model performance and calibration statistics (e.g., AUC, Hosmer–Lemeshow) are detailed in Supplementary Materials Section E, Table S8.

**Table 4:** Independent Predictors of Three Forms of Clinical Inertia: Device-Level (DLI), Therapeutic Class (TCI), and Adherence-Related (ARI).

Predictor	B (SE)	Wald χ^2^	df	aOR	95% CI	*p*
**A. Therapeutic Class Inertia (TCI)—High-Risk Patients (n = 335)**						
Phenotype (Cluster 2 vs. 1)	−0.34 (0.67)	0.27	1	0.71	0.18–2.72	0.613
Age (per 10-year increase)	−0.51 (0.13)	14.9	1	0.60	0.46–0.77	<0.001 ***
Charlson Comorbidity Index (per point)	0.64 (0.21)	8.75	1	1.90	1.24–2.91	0.003 **
Baseline Therapy Potency (per level)	2.05 (0.39)	28.0	1	7.80	3.65–16.64	<0.001 ***
Admitting Service (Ref: Internal Medicine)						
Pulmonology	0.10 (0.83)	0.02	1	1.10	0.26–4.61	0.891
Other	1.77 (0.82)	4.67	1	5.88	1.17–29.44	0.031 *
**B.1. Device-Level Inertia (DLI)—Baseline Model (n = 114)**						
Phenotype (Cluster 2 vs. 1)	−1.84 (1.05)	3.07	1	0.16	0.02–1.24	0.080
Age (per 10-year increase)	−0.58 (0.33)	3.18	1	0.56	0.30–1.06	0.075
Charlson Comorbidity Index (per point)	−0.08 (0.12)	0.43	1	0.92	0.73–1.17	0.513
Admitting Service (Ref: Internal Medicine)						
Pulmonology	−1.46 (0.76)	3.67	1	0.23	0.05–1.03	0.055
Other	−0.99 (1.33)	0.55	1	0.37	0.03–5.02	0.457
Respiratory Disease (Ref: None)						
COPD	1.51 (1.13)	1.80	1	4.54	0.50–41.50	0.180
Asthma	1.80 (1.00)	3.23	1	6.07	0.85–43.31	0.072
Bronchiectasis	0.66 (1.71)	0.15	1	1.93	0.07–54.86	0.702
Pre-admission Device (Ref: pMDI/SMI)						
DPI	0.30 (0.85)	0.13	1	1.35	0.25–7.23	0.722
Spacer	0.56 (0.83)	0.45	1	1.75	0.35–8.84	0.500
**B.2. Device-Level Inertia (DLI)—Expanded Model with Training Variables (n = 101)**						
Age (per 10-year increase)				1.06	0.74–1.51	0.760
Charlson (per point)				0.99	0.81–1.20	0.900
Service: Pulmonology (vs. Internal Medicine)				1.00	0.25–4.00	1.000
Service: Other (vs. Internal Medicine)				1.00	0.25–4.00	1.000
Pre-admission device: DPI (vs. pMDI/SMI)				1.26	0.54–2.97	0.596
Pre-admission device: Spacer (vs. pMDI/SMI)				1.36	0.59–3.16	0.475
In-hospital device: SMI only (vs. Nebulizer only)				0.31	0.09–1.05	0.061
In-hospital device: pMDI only (vs. Nebulizer only)				1.00	0.46–2.17	1.000
In-hospital device: Mixed (vs. Nebulizer only)				1.00	0.29–3.39	1.000
In-hospital device: DPI/Spacer only (vs. Nebulizer only)				1.64	0.49–5.46	0.418
Any inhaler training documented (vs. none/unknown)				1.41	0.59–3.40	0.439
Trainer = Pharmacy or Specialist				1.00	0.41–2.42	1.000
Training rating: Poor/Very poor				1.00	0.37–2.70	1.000
Training: Did not receive				3.49	1.21–10.03	0.020 *
**C. Adherence-Related Inertia (ARI)—Patients with Documented Poor Adherence (n = 106)**						
Phenotype (Cluster 2 vs. 1)	1.19 (0.80)	2.22	1	3.29	0.67–16.14	0.142
Age (per 10-year increase)	0.26 (0.18)	2.18	1	1.29	0.92–1.82	0.140
Charlson Comorbidity Index (per point)	–0.14 (0.15)	0.90	1	0.87	0.65–1.16	0.342
Home Maintenance Inhalers (per device)	–2.81 (0.65)	18.7	1	0.06	0.02–0.25	<0.001 ***

Note. Values are adjusted odds ratios (aORs) with 95% confidence intervals (CI), and corresponding regression coefficients (B), standard errors (SEs), Wald χ^2^ tests, and *p*-values. Each model was fitted using multivariable logistic regression within its relevant patient denominator. TCI analyses included only high-risk patients (n = 335). DLI analyses were restricted to patients with a documented critical error; the baseline model included n = 114, and the expanded model included n = 101 due to missing data on training variables. ARI analyses included only patients with documented poor adherence (n = 106). An aOR > 1 indicates higher odds of inertia; an aOR < 1 indicates lower odds. Abbreviations: ARI = Adherence-Related Inertia; CI = confidence interval; DLI = Device-Level Inertia; TCI = Therapeutic Class Inertia. Significance thresholds: * *p* < 0.05; ** *p* < 0.01; *** *p* < 0.001; Model performance and calibration statistics (e.g., AUC, Hosmer–Lemeshow) are detailed in Supplementary Materials Section E, Table S8.

**Table 5:** Multivariable Logistic Regression for Device Change at Discharge.

Predictor	Adjusted Odds Ratio (aOR)	95% CI	*p*-Value
Age (per 10-year increase)	0.89	0.76–1.04	0.148
Charlson Comorbidity Index (per point)	0.96	0.87–1.05	0.333
Service (Ref: Internal Medicine)			
Pulmonology	1.00	0.25–4.00	1.000
Other	0.88	0.22–3.51	0.853
Pre-admission device (Ref: DPI)			
pMDI/SMI	1.85	1.21–2.82	0.004 **
Spacer	1.00	0.58–1.72	1.000
In-hospital device (Ref: Nebulizer only)			
SMI only	2.79	1.10–7.09	0.031 *
pMDI only	1.02	0.68–1.52	0.926
Mixed	1.00	0.35–2.82	1.000
DPI only/Spacer only	0.22	0.07–0.68	0.009 **

Note. Values are adjusted odds ratios (aORs) with 95% confidence intervals (CI). An aOR > 1 indicates higher odds of a device change; an aOR < 1 indicates lower odds. The model was fitted on N = 499 patients. Abbreviations: CI, confidence interval; DPI, dry powder inhaler; pMDI, pressurized metered-dose inhaler; SMI, soft mist inhaler. * *p* < 0.05, ** *p* < 0.01

## Data Availability

The original contributions presented in the study are included in the article; the data presented in this study are available on request from the corresponding author.

## Supplementary Materials

The following supporting information can be downloaded at: https://www.mdpi.com/article/10.3390/biomedicines13122892/s1, Table S1: Logistic Regression of CFCS on Adverse Outcomes, Stratified by Phenotype; Table S2: Unadjusted Association Between Non-Adherence Domains and Critical Inhaler Errors; Table S3: Association Between Functional Capacity and Adverse Outcomes, Stratified by Clinical Phenotype; Table S4: Flow of Patient Selection for the Analytical Cohort; Table S5: Proportion of Missing Data for Key Variables; Table S6: Power Assessment and Multicollinearity Diagnostics; Table S7: Cluster Validation and Sensitivity Analysis; Table S8: Performance of Primary Multivariable Logistic Regression Models; Table S9: Key Interaction Analyses for Effect Modification by Phenotype; Table S10: Associations of Composite Functional Capacity Score (CFCS) With Clinical Outcomes Across Main and Sensitivity Analyses; Figure S1: Deviations in Clinical Inertia Prevalence by Subgroup and Phenotype; Figure S2: Interaction of Phenotype with Clinical Factors on the Predicted Probability of Adherence-Related Inertia and Critical Errors; Figure S3: Predicted probabilities of clinical outcomes by composite functional capacity score (CFCS).

## Abbreviations

The following abbreviations are used in this manuscript:

TCI	Therapeutic Class
DLI	Device-Level
ARI	Adherence-Related
CFCS	Clinical Frailty and Competence Score
TAI	Test of Adherence to Inhalers
DPI	Dry powder inhaler
pMDI	Pressurized metered-dose inhaler
SMI	Soft mist inhaler
