# Supplementary material for: Deconstructing Therapeutic Failure with Inhaled Therapy in Hospitalized Patients: Phenotypes, Risk Profiles, and Clinical Inertia
Source: Biomedicines. 2025 Nov 26;13(12):2892. doi: 10.3390/biomedicines13122892 (PMC12731047; doi:10.3390/biomedicines13122892)
Supplement: Supplementary file 1 [file biomedicines-13-02892-s001.zip › biomedicines-3947577-File S2.pdf]

Methodological Analysis

A. Study Population and Patient Selection

This study employed a consecutive enrollment strategy to minimize selection bias. All adult patients admitted to medical inpatient services prescribed any form of inhaled therapy between March 2023 and March 2024 were prospectively screened for eligibility. The table below summarizes the flow of participants, demonstrating that the final analytical cohort of 499 patients represents a well-defined and systematically selected population from the source cohort.

Table S4. Flow of Patient Selection for the Analytical Cohort.

Screening and Enrollment Phase	Number of Patients
Patients admitted and on inhaled therapy screened for eligibility	985
**Excluded (n=486)**	
• No documented history of *chronic* inhaled therapy	251
• Severe cognitive impairment precluding consent/assessment	165
• Incomplete in-hospital assessment or missing discharge data	48
• Patient refusal to provide informed consent	22
**Final Analytical Cohort**	499

Note. This systematic screening and application of pre-specified eligibility criteria ensure the internal validity of the cohort.

B. Handling of Missing Data

The study was designed for prospective and complete data collection. For the final analysis, a complete-case approach was adopted, an appropriate strategy given the very low proportion of missing data (<5%) for all key analytical variables. This high level of data completeness minimizes the risk of bias that could arise from imputation techniques.

Table S5. Proportion of Missing Data for Key Variables.

Variable	Proportion Missing (%)
90-Day Mortality	0.0%
Peak Inspiratory Flow (PIF)	1.2%*
TAI Adherence Score	0.8%
Handling Knowledge	0.4%
Charlson Comorbidity Index	0.0%

Note. Missing data proportions were minimal. *The "Unknown" category for Peak Flow (36.5%) was treated as a distinct, clinically meaningful analytical category representing the failure to assess, not as missing data.

C. Study Power and Model Diagnostics

A post-hoc power analysis confirmed the adequacy of the 499-patient cohort. The criterion of at least 10 events per variable (EPV) was met for all multivariable models, ensuring sufficient power. To guarantee the stability of the regression coefficients, all models were assessed for multicollinearity using the Variance Inflation Factor (VIF). All VIF values were well below the concern threshold of 5, confirming the absence of significant collinearity.

Table S6. Power Assessment and Multicollinearity Diagnostics.

Model Outcome	Events Per Variable (EPV)	Mean VIF	Max VIF
90-Day Mortality	12.0	1.29	1.48
Critical Inhaler Errors	16.4	1.15	1.25
Therapeutic Class Inertia (TCI)	37.8	1.21	1.33
Device-Level Inertia (DLI) – Baseline (n = 114)	12.7	1.18	1.32
Device-Level Inertia (DLI) – Expanded (n = 101)	10.1	1.20	1.36

D. Phenotype Identification and Validation

The two-phenotype structure was empirically validated using average silhouette scores, with the two-cluster solution showing a strong score of 0.68. Importantly, outcome variables were not used in the clustering algorithm to avoid circular reasoning. A sensitivity analysis using multiple imputation confirmed the stability of the phenotypes (97.2% agreement), demonstrating that the "unassessed" status is a robust clinical characteristic.

Table S7. Cluster Validation and Sensitivity Analysis.

Analysis	Metric	Result
**Cluster Validation**	Number of Clusters (k)	Average Silhouette Width
	2	0.68
	3	0.51
**Phenotype Stability**	Mean Patient Re-classification Agreement	97.2%

E. Model Performance and Calibration

The performance of the primary predictive models was assessed for discrimination (area under the receiver operating characteristic curve - AUC) and calibration (Hosmer-Lemeshow test). As shown in Table E1, the models for mortality and critical errors demonstrated excellent discrimination and calibration. For Device-Level Inertia, the baseline model showed poor discrimination, which was substantially improved in the expanded model that included process-of-care variables.

Table S8. Performance of Primary Multivariable Logistic Regression Models.

Model Outcome	Discrimination (AUC)	95% Confidence Interval	Calibration (Hosmer–Lemeshow p-value)
90-Day Mortality	0.86	0.82 – 0.90	0.45
Critical Inhaler Errors	0.83	0.78 – 0.88	0.51
Device-Level Inertia (DLI) – Baseline	0.60	0.52 – 0.68	0.21
Device-Level Inertia (DLI) – Expanded	0.73	0.65 – 0.81	0.34

Note. AUC values > 0.80 indicate excellent discrimination. AUC values between 0.70-0.80 indicate acceptable discrimination, while values below 0.60 indicate poor discrimination. Hosmer-Lemeshow test p-values > 0.05 indicate good model calibration.

F. Supplementary Analyses of Therapeutic Failure

Unadjusted analyses confirmed that non-adherence was significantly associated with critical inhaler errors. Furthermore, an analysis of effect modification revealed significant statistical interactions between phenotype and key risk factors (e.g., adherence), validating a stratified analytical approach.

Table S9. Key Interaction Analyses for Effect Modification by Phenotype.

Outcome	Interaction Term	OR (95% CI)	*p* for Interaction
**Critical Errors**	TAI sum (adherence) × Phenotype	0.86 (0.74–0.99)	0.048
	Hosp/ED visits × Phenotype	1.54 (1.02–2.33)	0.040
**Adherence-Related Inertia**	Peak flow (L/min) × Phenotype	1.07 (1.01–1.14)	0.033

G. Construction and Validation of the Composite Functional Capacity Score (CFCS)

The Composite Functional Capacity Score (CFCS) was created to provide an integrated measure of a patient's capability to use their inhaler device correctly. It was constructed by averaging three rescaled domains: (a) peak inspiratory flow, (b) a knowledge composite, and (c) TAI adherence. Higher CFCS values indicate greater functional capacity for inhaler use.

The robustness of the CFCS as a predictor was confirmed through sensitivity analyses, with detailed results presented in Table G1. These analyses included testing a worst-case scenario for missing knowledge data and recalculating effect sizes per interquartile range. Across all specifications, the CFCS remained a strong and consistent predictor of process outcomes, particularly device-level inertia and critical errors, underscoring its validity. Figure G1 provides a visual representation of these modeled associations.

Table S10. Associations of Composite Functional Capacity Score (CFCS) With Clinical Outcomes Across Main and Sensitivity Analyses.

Outcome	Coding	Main model aOR (95% CI) per +0.1 / +1 SD	p	Sensitivity A (Unknown=0) aOR (95% CI)	p	IQR (units)	Sensitivity B aOR (95% CI) per +1 IQR	p
**Device-Level Inertia (new rule)**	CFCS_div30	0.85 (0.80–0.91)	< .001	0.11 (0.05–0.24)	< .001	0.02	0.79 (0.74–0.84)	< .001
	CFCS_z	0.70 (0.61–0.80)	< .001	0.32 (0.19–0.55)	< .001	0.395	0.64 (0.55–0.73)	< .001
**Therapeutic Class Inertia (high-risk only)**	CFCS_div30	1.00 (0.94–1.06)	.940	0.61 (0.31–1.21)	.155	0.02	0.98 (0.92–1.04)	.472
	CFCS_z	0.99 (0.88–1.12)	.860	1.05 (0.68–1.62)	.820	0.395	0.98 (0.87–1.10)	.694
**Adherence-Related Inertia (poor adherence)**	CFCS_div30	1.08 (0.97–1.21)	.160	1.50 (0.96–2.35)	.073	0.02	1.12 (1.01–1.25)	.038
	CFCS_z	1.04 (0.90–1.20)	.590	1.18 (0.77–1.82)	.449	0.395	1.09 (0.92–1.28)	.315
**Critical Errors**	CFCS_div30	0.53 (0.42–0.68)	< .001	0.07 (0.03–0.15)	< .001	0.07	0.46 (0.36–0.60)	< .001
	CFCS_z	0.41 (0.29–0.57)	< .001	0.15 (0.09–0.26)	< .001	0.975	0.29 (0.20–0.42)	< .001
**90-day Mortality (discharged alive)**	CFCS_div30	1.00 (0.90–1.11)	.980	1.02 (0.43–2.18)	.940	0.03	1.01 (0.91–1.11)	.920
	CFCS_z	0.98 (0.86–1.12)	.750	0.97 (0.74–1.29)	.858	0.400	0.97 (0.85–1.11)	.656

Note. Odds ratios (ORs) estimated from logistic regression models adjusted for age, sex, phenotype, Charlson comorbidity index, and admitting service. Main models tested two codings: CFCS_div30 (scaled per +0.1 increment) and CFCS_z (standardized per +1 SD). Sensitivity A recoded missing knowledge as 0 (worst-case). Sensitivity B expressed effects per interquartile range (IQR). Bolded values indicate statistical significance at $p < 0.05$.

Figure S3. Predicted probabilities of clinical outcomes by composite functional capacity score (CFCS).

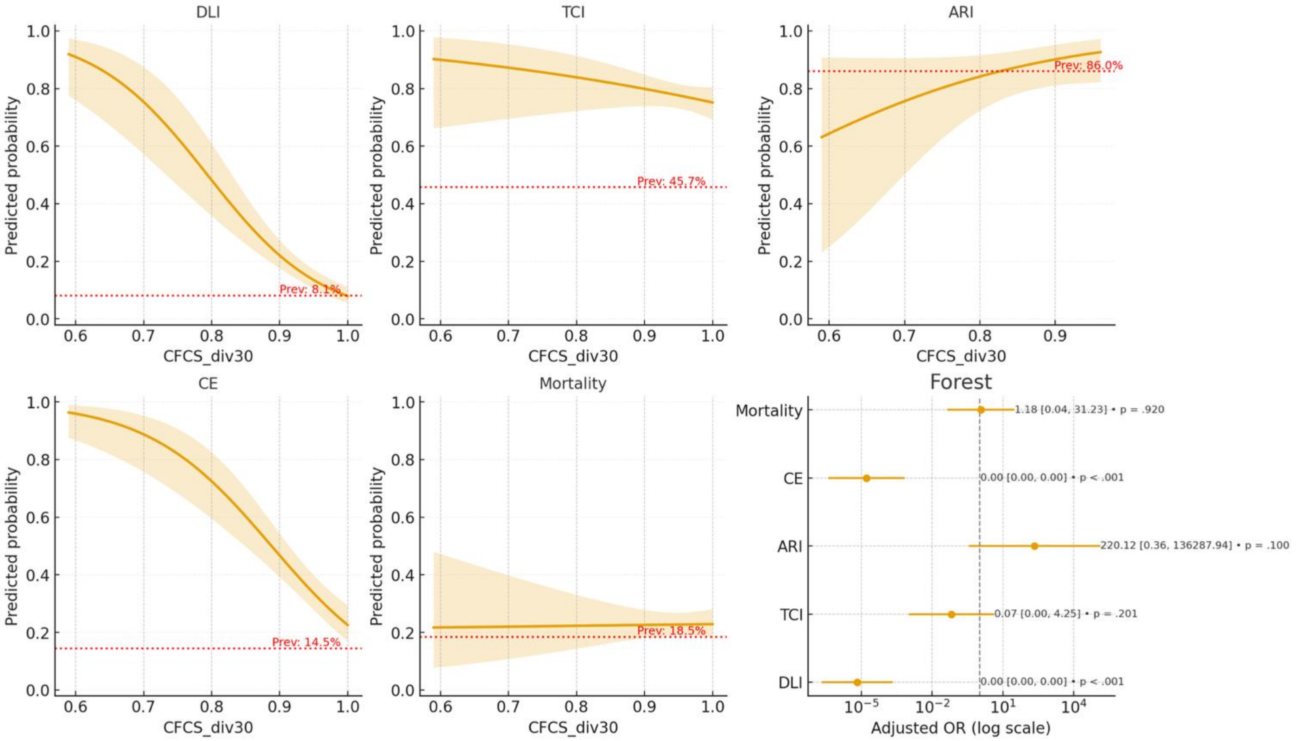

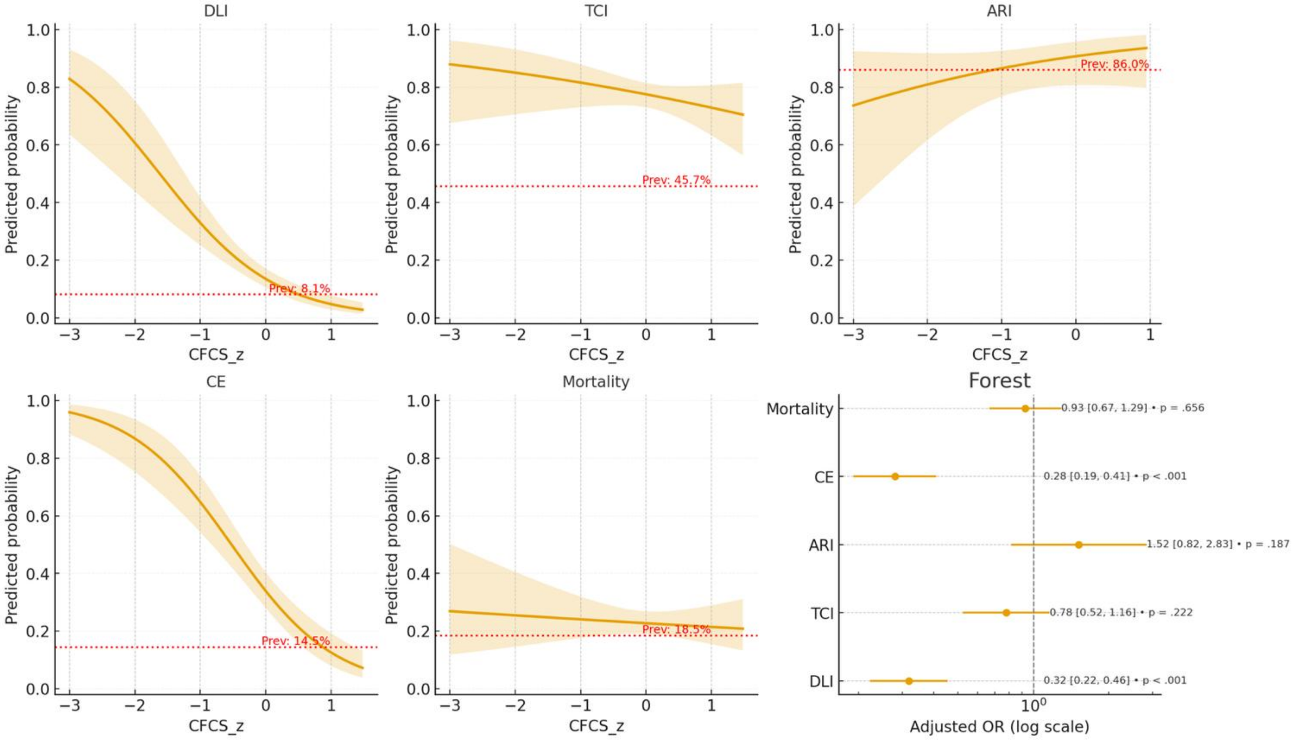

Note. Predicted probabilities (solid lines with shaded 95% CIs) are shown for Device-Level Inertia (DLI), Therapeutic Class Inertia (TC), Adherence-Related Inertia (ARI), Critical Errors (CE), and 90-day post-discharge mortality (Mortality). Panels compare CFCS estimated per +0.1 increase in the div30 version (top row) and per +1 SD in the z-score version (bottom row). The rightmost panels display adjusted odds ratios (forest plots) for each outcome, controlling for age and Charlson comorbidity index. Red dotted lines indicate outcome-specific prevalence in the study cohort.
