# Supplementary material for: Deconstructing Therapeutic Failure with Inhaled Therapy in Hospitalized Patients: Phenotypes, Risk Profiles, and Clinical Inertia
Source: Biomedicines. 2025 Nov 26;13(12):2892. doi: 10.3390/biomedicines13122892 (PMC12731047; doi:10.3390/biomedicines13122892)
Supplement: Supplementary file 1 [file biomedicines-13-02892-s001.zip › biomedicines-3947577-File S1.pdf]

Supplementary Table S1. Logistic Regression of CFCS on Adverse Outcomes, Stratified by Phenotype

Outcome	Cluster	B (SE)	Wald χ^2	df	OR (per +0.1 CFCS)	95% CI	p-value	Interpretation
**CE**	Cluster 1 (Unassessed/Older–Higher Burden)	−0.35 (3.05)	0.01	1	0.97	0.53–1.76	.909	No association.
	Cluster 2 (Assessed/Better-Documented)	−13.40 (2.38)	31.76	1	0.26	0.16–0.42	< .001	Strong protective effect: ~74% lower odds per +0.1 CFCS.
**TCI**	Cluster 1	−1.86 (2.42)	0.59	1	0.83	0.52–1.33	.443	No significant association.
	Cluster 2	0.80 (1.90)	0.18	1	1.08	0.75–1.57	.674	No significant association.
**DLI**	Cluster 1	−14.00 (3.12)	20.16	1	0.25	0.13–0.45	< .001	~75% lower odds per +0.1 CFCS.
	Cluster 2	−9.46 (2.06)	21.10	1	0.39	0.26–0.58	< .001	~61% lower odds per +0.1 CFCS.
**ARI**	Cluster 1	−22.64 (4.49)	25.48	1	0.10	0.04–0.25	< .001	~90% lower odds per +0.1 CFCS.
	Cluster 2	−20.60 (2.98)	47.75	1	0.13	0.07–0.23	< .001	Robust protection.
**Mortality**	Cluster 1	5.00 (3.27)	2.34	1	1.65	0.87–3.13	.126	Non-significant upward trend.
	Cluster 2	−3.59 (2.03)	3.13	1	0.70	0.47–1.04	.077	Borderline protective trend.

Note: Logistic regression models stratified by phenotype. CFCS predictor scaled 0–1; results presented per +0.1 CFCS increment. OR < 1.00 indicates protective association. Wald χ^2 from likelihood-ratio tests (df = 1). Significance thresholds: *p < 0.05; **p < 0.01; ***p < 0.001. Abbreviations: CFCS = Composite Functional Capacity Score; CE = Clinical Errors; TCI = Therapeutic Class Inertia; DLI = Device-Level Inertia; ARI = Adherence-Related Inertia.

Supplementary Table S1 shows the associations of CFCS with adverse outcomes across phenotypes, corresponding to the patterns illustrated in Figure 4. In Cluster 1, CFCS had no significant association with any of the adverse outcomes. In contrast, in Cluster 2 a higher CFCS was strongly protective against Clinical Errors (CE), Device-Level Inertia (DLI), and Adherence-Related Inertia (ARI), indicating a lower likelihood of these outcomes in patients with better functional status. For Therapeutic Class Inertia (TCI), CFCS had no discernible influence in either phenotype. For mortality, there was a non-significant upward trend in Cluster 1 and a borderline protective trend in Cluster 2 associated with higher CFCS.

Supplementary Table S2. Unadjusted Association Between Non-Adherence Domains and Critical Inhaler Errors

Domain	Crude Odds Ratio (Yes vs. No)	95% CI	*p*-value
Erratic non-adherence	1.69	0.99–2.88	0.052
Deliberate non-adherence	5.32	2.41–11.74	<0.001
Unintentional non-adherence	15.19*	(Haldane–Anscombe corrected)	0.005

Note: OR = odds ratio; CI = confidence interval. Unadjusted logistic regression models were used. *The Haldane–Anscombe correction was applied for unintentional non-adherence due to zero counts in one comparison cell. An OR > 1 indicates higher odds of a critical error.

Supplementary Table S2 revealed that all non-adherence domains were associated with an increased risk of critical inhaler errors. Unintentional non-adherence showed the most pronounced effect, with a dramatic fifteenfold increase in the odds of misuse. Deliberate non-adherence was associated with a more than fivefold increase in the odds of a critical error. Finally, erratic non-adherence was associated with a nearly 70% increase in the odds of an error, an effect that approached statistical significance.

Supplementary Figure S1. Deviations in Clinical Inertia Prevalence by Subgroup and Phenotype

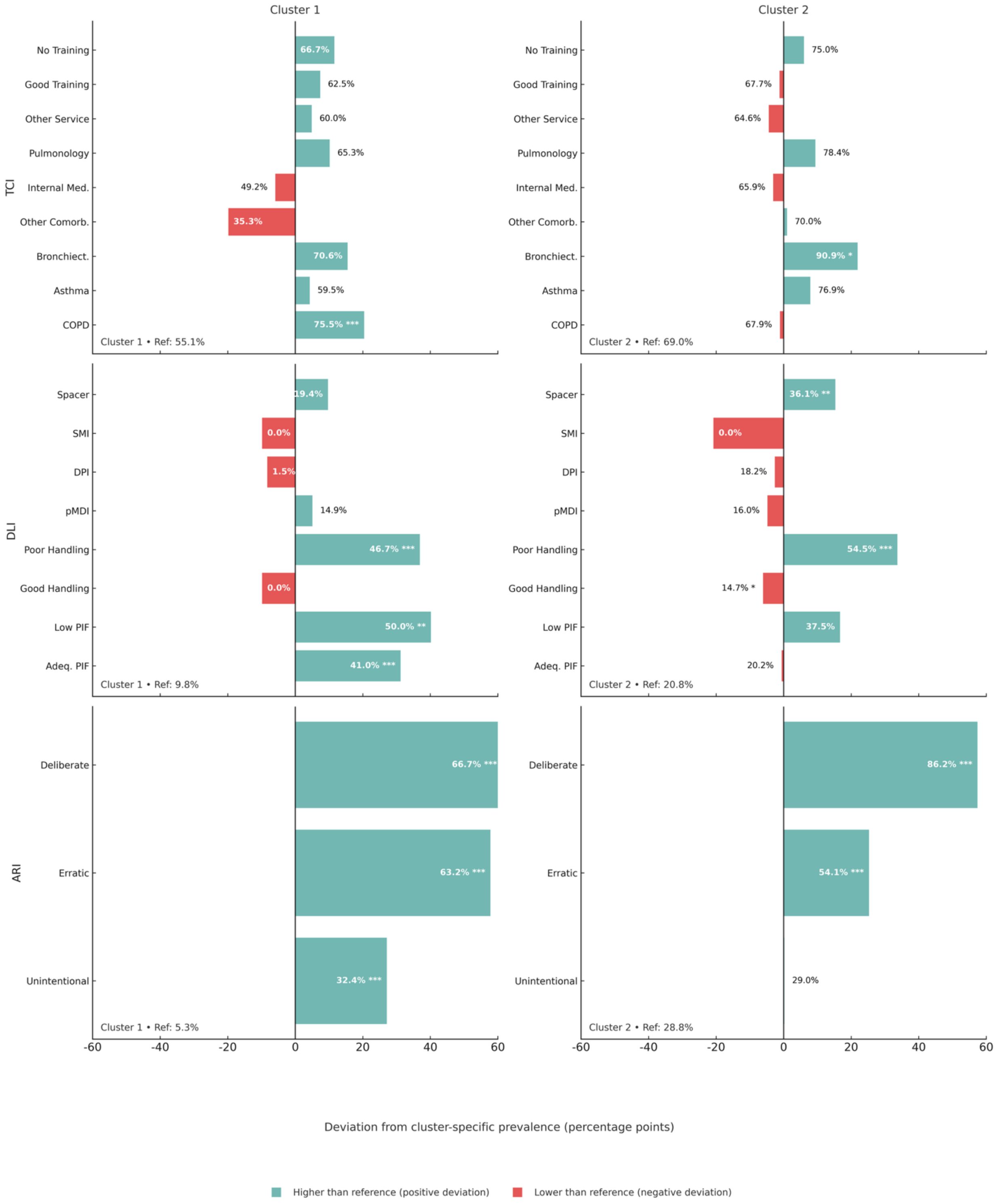

Note: Bar length represents the deviation in clinical inertia prevalence from the cluster-specific reference rate (percentage points), while bar labels indicate the absolute prevalence (%) within each subgroup. Subgroup sample

sizes are shown on the y-axis (n). Positive bars denote higher prevalence than the cluster reference, negative bars denote lower prevalence. Significance is denoted as: *$p < 0.05$; **$p < 0.01$; ***$p < 0.001$.

Supplementary Figure S1 shows that subgroup deviations from the baseline prevalence of clinical inertia varied by inertia type. For Therapeutic Class Inertia (TCI) and Adherence-Related Inertia (ARI), most deviations were modest. In contrast, for Device-Level Inertia (DLI), several subgroups showed statistically significant deviations from their cluster's baseline. Notably, in Cluster 1, Poor Handling and both Low and Adequate PIF were associated with significantly higher DLI prevalence. In Cluster 2, Spacer use was associated with a significantly higher prevalence of DLI, while Poor and Good Handling, as well as Low PIF, also showed significant deviations.

Supplementary Table S3. Association Between Functional Capacity and Adverse Outcomes, Stratified by Clinical Phenotype

Outcome	Cluster	Q1 % (n/N)	Q2 % (n/N)	Q3 % (n/N)	Δ% (Q3–Q1)	RR (Q3 vs Q1)	OR per +0.1 CFCS (95% CI)	Relative Composition (Q1→Q3)
**CE**	C1	76.9% (20/26)	82.0% (50/61)	80.0% (12/15)	+3.1% ↑	1.04	0.88 (0.71–1.08) ns	27.8% → 24.2%
	C2	76.2% (32/42)	80.3% (57/71)	77.8% (14/18)	+1.6% ↑	1.02	0.26 (0.14–0.48) ↓	13.2% → 11.5%
**TCI**	C1	42.3% (11/26)	55.7% (34/61)	53.3% (8/15)	+11.0% ↑	1.26	1.05 (0.82–1.35) ns	61.1% → 24.2%
	C2	50.0% (21/42)	63.4% (45/71)	61.1% (11/18)	+11.1% ↑	1.22	0.95 (0.72–1.25) ns	27.6% → 18.0%
**DLI**	C1	15.4% (4/26)	16.4% (10/61)	6.7% (1/15)	–8.7% ↓	0.43	0.25 (0.14–0.44) ↓	38.9% → 30.3%
	C2	16.7% (7/42)	15.5% (11/71)	22.2% (4/18)	+5.5% ↑	1.33	0.40 (0.23–0.70) ↓	52.6% → 18.0%
**ARI**	C1	3.8% (1/26)	1.6% (1/61)	6.7% (1/15)	+2.9% ↑	1.76	0.10 (0.04–0.22) ↓	5.6% → 3.0%
	C2	14.3% (6/42)	19.7% (14/71)	22.2% (4/18)	+7.9% ↑	1.55	0.13 (0.05–0.32) ↓	7.9% → 6.6%
**Mortality**	C1	23.1% (6/26)	39.3% (24/61)	20.0% (3/15)	–3.1% ↓	0.87	1.65 (0.86–3.16) ns	33.3% → 9.1%
	C2	33.3% (14/42)	18.3% (13/71)	16.7% (3/18)	–16.6% ↓	0.50	0.70 (0.47–1.04) ns	18.4% → 4.9%

Note. CFCS was divided into three quantile-based bins (Q1–Q3). Δ% is the absolute change in prevalence between Q3 and Q1; RR is the relative risk (Q3 vs Q1). Logistic regression models estimated adjusted odds ratios (OR) per +0.1 CFCS, with 95% confidence intervals (CI). ns = non-significant. Abbreviations: ARI, Adherence-Related Inertia; CE, Clinical Errors; CFCS, Composite Clinical Frailty and Competence Score; CI, Confidence Interval; DLI, Device-Level Inertia; TCI, Therapeutic Class Inertia.

This table provides a detailed numerical breakdown of the five adverse outcomes for each of the two clinical phenotypes, stratified by quantiles of the Composite Clinical Frailty and Competence Score (CFCS). The data presented here serve as the foundation for the visualizations in Figure 3 and the odds ratios in Figure 4.

Supplementary Figure S2. Interaction of Phenotype with Clinical Factors on the Predicted Probability of Adherence-Related Inertia and Critical Errors

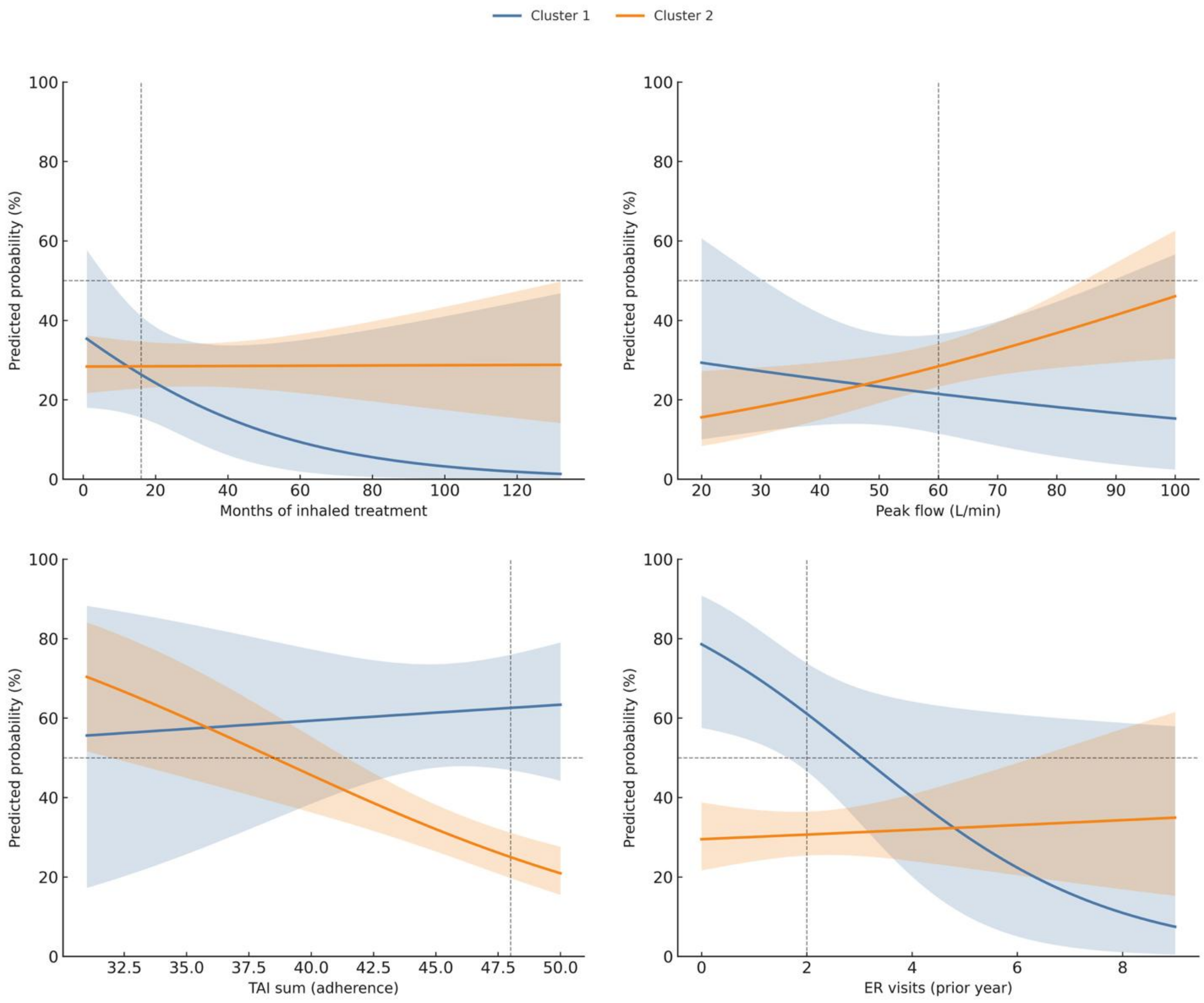

Note. Curves represent the predicted probability of the outcome based on fitted logistic regression models that include an interaction term between the clinical factor and the phenotype. Shaded areas represent 95% confidence intervals. Abbreviations: TAI, Test of Adherence to Inhalers; ED, Emergency Department.

Supplementary figure S2 illustrates four significant interaction effects, showing that the relationship between clinical factors and adverse outcomes differs between the two phenotypes.

The predictors of Adherence-Related Inertia (ARI) showed contrasting effects depending on the phenotype. For the "Unassessed/Older" phenotype (Cluster 1), both higher peak flow and a longer duration of inhaled treatment were associated with a lower probability of ARI. These protective effects were absent or even reversed in the "Assessed/Younger" phenotype (Cluster 2) (p for interaction = 0.033 and p = 0.016, respectively).

Similarly, the drivers of Critical Errors (CE) were phenotype-dependent. In the "Assessed/Younger" group, better self-reported adherence was strongly protective against errors,

a relationship that was not present in the "Unassessed/Older" group (p for interaction = 0.052). Conversely, a history of more frequent healthcare utilization (hospital or emergency department visits) was associated with a lower probability of critical errors, an effect seen only in the "Unassessed/Older" phenotype (p for interaction = 0.040).

While the preceding models examined the predictors of clinical inaction, we next sought to invert the analytical question. Instead of asking what drives inertia, we investigated what predicts positive action. Specifically, we modeled the factors associated with a device change at discharge. This complementary, or "mirror," analysis allows for a more complete understanding by revealing whether the factors that successfully prompt a change are distinct from those associated with the failure to act.
